# No perceptual prioritization of non-nociceptive vibrotactile and visual stimuli presented on a sensitized body part

**DOI:** 10.1038/s41598-018-23135-6

**Published:** 2018-03-29

**Authors:** D. M. Torta, L. Filbrich, E. N. Van Den Broeke, V. Legrain

**Affiliations:** 1 Institute of Neuroscience, Université catholique de Louvain, Brussels, Belgium; 2 Research group in Health Psychology, University of Leuven, Leuven, Belgium; 3 Psychological Sciences Research Institute, Université catholique de Louvain, Louvain-la-Neuve, Belgium

## Abstract

High frequency electrical conditioning stimulation (HFS) is an experimental method to induce increased mechanical pinprick sensitivity in the unconditioned surrounding skin (secondary hyperalgesia). Secondary hyperalgesia is thought to be the result of central sensitization, i.e. increased responsiveness of nociceptive neurons in the central nervous system. Vibrotactile and visual stimuli presented in the area of secondary hyperalgesia also elicit enhanced brain responses, a finding that cannot be explained by central sensitization as it is currently defined. HFS may recruit attentional processes, which in turn affect the processing of all stimuli. In this study we have investigated whether HFS induces perceptual biases towards stimuli presented onto the sensitized arm by using Temporal Order Judgment (TOJ) tasks. In TOJ tasks, stimuli are presented in rapid succession on either arm, and participants have to indicate their perceived order. In case of a perceptual bias, the stimuli presented on the attended side are systematically reported as occurring first. Participants performed a tactile and a visual TOJ task before and after HFS. Analyses of participants’ performance did not reveal any prioritization of the visual and tactile stimuli presented onto the sensitized arm. Our results provide therefore no evidence for a perceptual bias towards tactile and visual stimuli presented onto the sensitized arm.

## Introduction

Cutaneous tissue injury induces increased pain sensitivity (hyperalgesia) within the injured area (“primary” hyperalgesia) and also in the surrounding non-injured skin (“secondary” hyperalgesia). Secondary hyperalgesia is thought to be the result of central sensitization, defined by the International Association for the Study of Pain (IASP) as “*an increased responsiveness of nociceptive neurons in the central nervous system to their normal or subthreshold afferent input”*^1^. Secondary hyperalgesia can also be induced experimentally, for example, after capsaicin treatment^2^ or by high frequency electrical stimulation (HFS), which consists of five 1-second trains of painful electrical stimuli delivered on the skin^3,4^. By using these methods, studies have showed that secondary hyperalgesia is characterized by increased pain sensitivity to mechanical pinprick stimuli but not heat stimuli^5,6^; and by increased pinprick evoked potentials (PEPs)^7^.

Interestingly, previous studies have reported an increase in brain responses measured by event related potentials (ERPs) to non-nociceptive vibrotactile and visual stimuli presented into the area of secondary hyperalgesia^8,9^ 20 minutes after the end of HFS. This increase cannot be explained in terms of central sensitization as it is currently defined by the IASP^1^ (for a debate about the concept of central sensitization please refer to^10,11^) and may be considered a corollary HFS-induced effect involving supra-spinal cognitive mechanisms^9^, possibly attentional ones.

Attention allocated towards a particular stimulus or location typically leads to a *perceptual bias*, meaning that the processing of attended stimuli is speeded up at the detriment of unattended ones^12,13^. We hypothesized that HFS would increase attentional allocation towards the sensitized arm and consequently induce a perceptual bias towards the sensitized body part leading to a prioritization of the processing of the stimuli applied onto that body part. Indeed, if the aim of sensitization is to protect the body from further injury and promote healing, one might expect that, after inducing sensitization, all sensory stimuli presented on the sensitized body part could potentially constitute a threat to monitor. The possibility that HFS promotes the voluntary (endogenous) allocation of spatial attention towards the sensitized body location would also explain why after HFS the ERP vertex negative component (e.g. the negative peak recorded at approximately 150–200 ms at Cz, sometimes referred to as N1 or N2) elicited by sensory stimuli of several modalities was increased^8,9,14–17^. Indeed, previous studies have reported a larger vertex negativity in response to sensory stimuli presented within the focus of attention^18–23^. Our hypothesis, that HFS would prompt an endogenous attentional deployment towards the stimulated arm leading to a perceptual bias, was tested by using a temporal order judgment (TOJ) task, a well-established method to assess perceptual biases^12,13,24–26^.

In TOJ tasks, pairs of stimuli are presented in rapid succession at different temporal delays (i.e. at different SOAs for stimulus onset asynchronies between the stimuli of the pair) and participants have to indicate which of the two stimuli they perceived as presented first. Perceptual changes in TOJ tasks, reflected in the tendency to report the stimuli presented on one side as occurring before the stimuli presented on the other side, are interpreted in terms of *prior entry*. The priory entry law states that attended stimuli are perceived prior to unattended stimuli^12,25,27^, and that unattended stimuli have to be presented before attended stimuli in order to be perceived as simultaneous. In TOJ tasks, the Point of Subjective Simultaneity (PSS) is the parameter used to characterize prior entry. The PSS corresponds to the threshold of the psychometric function fitting the participants’ responses (defined in terms of probability of responding that one of the stimuli was perceived as first at each presented SOA), and characterizes the SOA at which the two stimuli have an equal chance of being perceived as presented first (for a review, see^25^). Shifts in PSS pinpoint changes in stimulus processing due to perceptual biases. We included in our study two TOJ tasks on pairs of vibrotactile stimuli applied onto the sensitized and control arm, and on pairs of visual stimuli flashed on either arm. We used an adaptive TOJ task, which has the advantage of selecting the SOAs between the two stimuli on the basis of the performance of the participant (Bayesian approach), thereby allowing a smaller number of trials^28,29^ without reducing the precision of the outcome (see^30^). Participants’ performance was measured immediately before HFS (T0) and twice after HFS, at 20 (T1) and at 45 (T2) minutes after the end of sensitization, in line with the experimental design used in previous studies (e.g.^16,31^). Perceptual biases were indexed by significant deviations of the PSS from 0 or significant changes across time points.

## Results

### Secondary hyperalgesia

**Figure 1:**
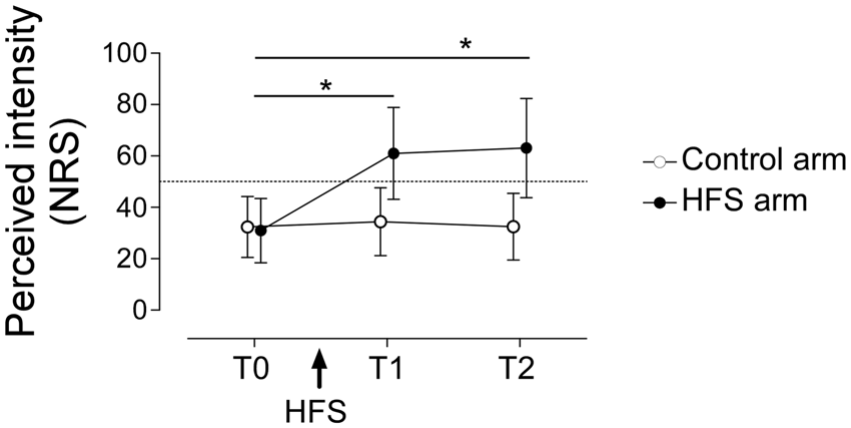
The perceived intensity of the pinprick stimuli was measured at each time point on a numerical rating scale (NRS) anchoring the value of 50 for the transition between the non-painful and painful sensations. The perception of mechanical pinprick stimuli was increased at T1 (20 minutes after HFS) and T2 (45 minutes after HFS) as compared to T0 for stimuli applied on the HFS, but not on the control arm. Asterisks denote a p < 0.001.

A pre-requisite of our experimental question was that HFS effectively induced secondary hyperalgesia, a manifestation of central sensitization. A first analysis showed that this was the case (see Fig. 1), as confirmed by the significant ‘*Time* * *Side*’ interaction (F(1,16) = 29.284 p < 0.001, η^2^_p_ = 0.647). Post-hoc t-tests revealed a significant increase in the perceived intensity of the stimuli applied onto the HFS arm (T0 vs. T1: t(16) = −6.559, p < 0.001; T0 vs. T2: t(16) = −6.550, p < 0.001), but not onto the control arm (T0 vs. T1: t(16) = −1.046, p = 0.311; T0 vs. T2: t(16) = −0.046, p = 0.964).

### TOJ tasks

Participants did not report any change in the perceived brightness of the visual stimuli or intensity of the vibrotactile stimuli across blocks and no difference between the two arms at any of the time points. Vibrotactile stimuli were never perceived as painful.

**Figure 2:**
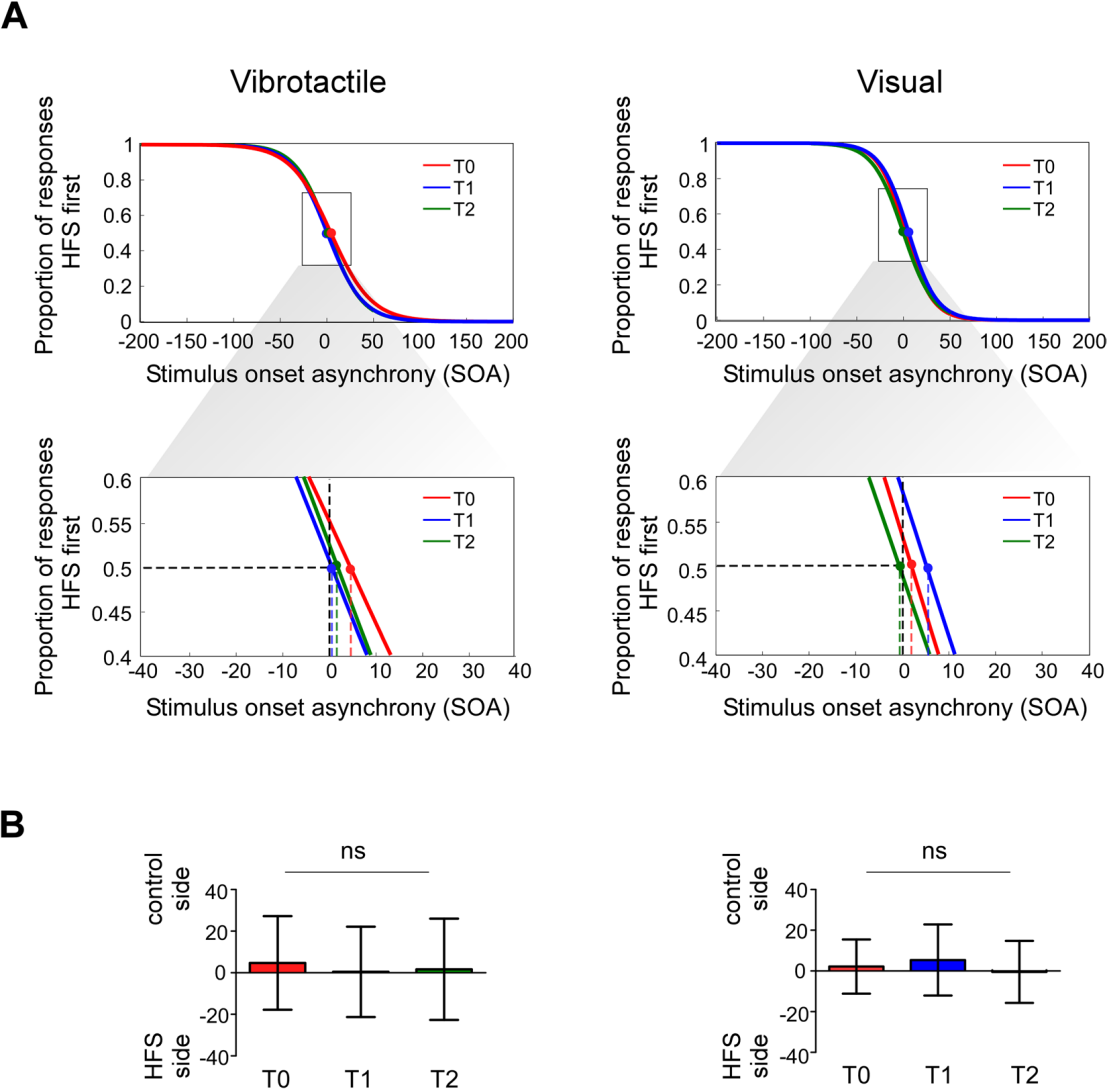
Panel A. Group level results. Data were fitted with a logistic function. The proportion of trials in which the stimulus applied onto the HFS arm was reported as occurring first was plotted as a function of the Stimulus Onset Asynchrony (SOA). Negative values indicate that the HFS side was stimulated first. The black dotted lines in the lower part of the panel correspond to the coordinates (x = 0; y = 0.5) representing the SOA at which the two stimuli have equal chance to be perceived first i.e. they represent the absence of any perceptual bias. For each time point, i.e. T0, T1 and T2, PSS values were compared against zero to disclose the presence of a perceptual bias. No significant shift emerged (i.e. none of the values was significantly different from zero). Panel B. Histograms represent PSS values. Upwards values show how many milliseconds the stimulus applied onto the control arm had to precede those applied onto the HFS arm to be perceived as occurring first.

T-tests did not reveal any significant difference from 0 for the PSS at any of the time points (all |t| < 1.302, all p > 0.210). Furthermore, PSS values did not change across time points, neither during the visual TOJ (F(1,17) = 1.715 p = 0.195 η^2^_p_ = 0.092), nor during the vibrotactile TOJ (F(1,17) = 0.809 p = 0.426 η^2^_p_ = 0.107). Similar results were observed for values characterizing the slope of the fitting functions (visual TOJ: F(1,17) = 0.547, p = 0.584, η^2^_p_ = 0.031; vibrotactile TOJ: F(1,17) = 0.356, p = 0.729, η^2^_p_ = 0.010). Data at the group level are illustrated in Fig. 2 (panel A).

We performed additional Bayesian analyses  to estimate the probability that HFS did not induce a significant perceptual bias, in other words to support the null-hypothesis H_0_. In contrast with frequentist statistics, which only allow the conclusion that there is no evidence supporting the alternative hypothesis H_1_ (in our case that HFS leads to a perceptual bias), Bayesian statistics can quantify the probability that H_0_ is true (i.e. that HFS does not lead to a perceptual bias^)^ as compared to H_1_ (i.e. that HFS leads to a perceptual bias)^32,33^.

The Bayesian one-sample t-tests supported the null hypothesis (i.e. bias absent) at all time points for the tactile (T0: BF_01_ = 2.924, BF_10_ = 0.342; T1: BF_01_ = 4.102, BF_10_ = 0.244; T2: BF_01_ = 3.964, BF_10_ = 0.252, *‘moderate evidence’*), and the visual TOJ (T0: BF_01_ = 3.338, BF_10_ = 0.300; T1: BF_01_ = 1.988, BF_10_ = 0.503; T2: BF_01_ = 4.081, BF_10_ = 0.245, *‘moderate evidence’*), although the evidence for the visual TOJ at T1 was less compelling (*‘anecdotal evidence’*; a graphical representation and the detailed statistics can be found in the supplementary file, and an explanation of the labels ‘moderate’ and ‘anecdotal’ can be found in the methods section). The Bayesian repeated measures ANOVA on the PSS values in the tactile TOJ provided evidence in support of the null model H_0_ (‘*Time’* BF_01_ = 3.911, BF_10_ = 0.256, *‘moderate evidence’*). The same analysis in the visual TOJ revealed the same tendency, but with less clear- cut results (‘*Time’* BF_01_ = 2.091, BF_10_ = 0.478, *‘anecdotal evidence’*).

Therefore, in order to investigate differences between the two modalities, we carried out a 3 × 2 Bayesian ANOVA, using the ‘*Time’* (T0, T1 and T2) and the ‘*Modality’* (Vibrotactile, Visual) as factors. The results strongly supported the null-model that HFS did not induce perceptual biases (Interaction ‘*Time* * *Modality’* BF_01_ = 10.777, BF_10_ = 0.024, *‘strong evidence’*, all the details of this analysis and the results of the same frequentist ANOVA can be found in the supplementary file).

## Discussion

In this study we have investigated whether HFS induced an endogenous attentional deployment towards the hyperalgesic arm leading to a perceptual bias towards the stimuli presented on that arm. A perceptual bias towards the HFS arm should have led participants to prioritize the stimuli applied on the sensitized arm, resulting in a PSS shift towards the control arm. We did not observe any significant change in PSS after HFS suggesting that HFS does not induce a perceptual bias to non-nociceptive stimuli delivered to the sensitized body part.

It is unlikely that our findings can be explained by a lack of sensitivity of the TOJ task. There is extensive literature showing that TOJ tasks can detect perceptual biases^12,13,25,28,34–42^. These biases can be generated by *exogenous* shifts of attention when lateralized non-target stimuli are presented shortly before the pair of target stimuli (e.g.^29,38,42^), but also by *endogenous* shifts of attention when participants voluntary allocate spatial attention towards a location, either in response to a cue pointing in that direction or due to increased motivation to attend to the location (e.g.^13,39–42^). In more detail, TOJ studies in several sensory domains have shown that unilateral non-target stimuli presented a few milliseconds before the pair of TOJ target stimuli bias the PSS towards the uncued side, i.e. they trigger exogenously a bias, leading to a prioritization of the stimuli presented on the cued side at the expense of the stimuli presented on the uncued side (e.g.^12,29,38,42–44^). Other studies have pointed out that endogenous attentional changes, like the ones we were expecting after HFS, can lead to significant perceptual biases towards the attended location or modality, resulting in a prioritization of stimuli presented at the attended body site (e.g.^13,24,39–42,45^). Taken together these results indicate that TOJ tasks are sensitive in detecting perceptual biases induced by exogenous and endogenous shifts in spatial attention. Notably, the lack of evidence for a perceptual bias in the present study for vibrotactile and visual stimuli does not exclude the possibility that there could be a perceptual bias for mechanical pinprick stimuli.

Does the lack of perceptual bias after HFS allow us to conclude that HFS does not influence spatial attention? Several studies have reported an enhancement of the vertex negativity for stimuli presented within the focus of spatial attention^18–23^ and the same increase was observed in previous HFS studies^8,9,14–17^. Therefore it is possible that HFS increases spatial attention towards the sensitized arm leading to a larger negative ERP peak, but without inducing any significant perceptual bias. Importantly, several attentional mechanisms and pathways have been characterized and their recruitment often depends on the kind of task and the relevance of the stimuli (for reviews in different sensory domains see^46–49^). A functional magnetic resonance study has evidenced that the execution of a tactile TOJ task activated the inferior parietal cortex, the superior and middle frontal gyri, and also the pre-motor cortices^50^. Instead, spatial attention directed to tactile or visual stimuli modulates activity in the corresponding sensory regions, the insula and the cingulate cortex besides the frontal-parietal attentional networks^51–57^. It is therefore possible that the two tasks do not engage the same cortical structures.

To conclude, we have found that HFS does not induce a perceptual bias towards non-nociceptive stimuli presented onto the HFS-treated arm. This suggests that the mechanism underlying the previously reported enhancement of brain responses elicited by non-nociceptive visual and vibrotactile stimuli is most likely different from the one mediating the prioritization of stimuli.

## Methods

### Participants

Eighteen healthy volunteers took part in the experiment (mean age 23.39, range 19–41, 7 men). Sample size was estimated on the basis of previous studies reporting an increase of the ERPs after HFS-induced sensitization (e.g.^14,16,58^). Exclusion criteria comprised having already participated in an HFS experiment, cardiac, neurologic and psychiatric complaints, history of chronic pain or ongoing pain, acute pain on the day of testing, usual intake of psychotropic medication, and intake of centrally active and analgesic drugs (e.g. NSAIDs) on the day of testing or on the day preceding the test. All participants were naïve to the aims of the study and were right handed according to the Flinders questionnaire^59^. The experimental procedure was approved by the local ethic committee (Commission d’Ethique Biomédicale Hospitalo-Facultaire de l’Université catholique de Louvain) in agreement with the Declaration of Helsinki and was carried out in accordance with the corresponding guidelines and regulations. All participants signed an informed consent form prior to the experimental session and received financial compensation for their participation.

### Stimuli and materials

**Figure 3:**
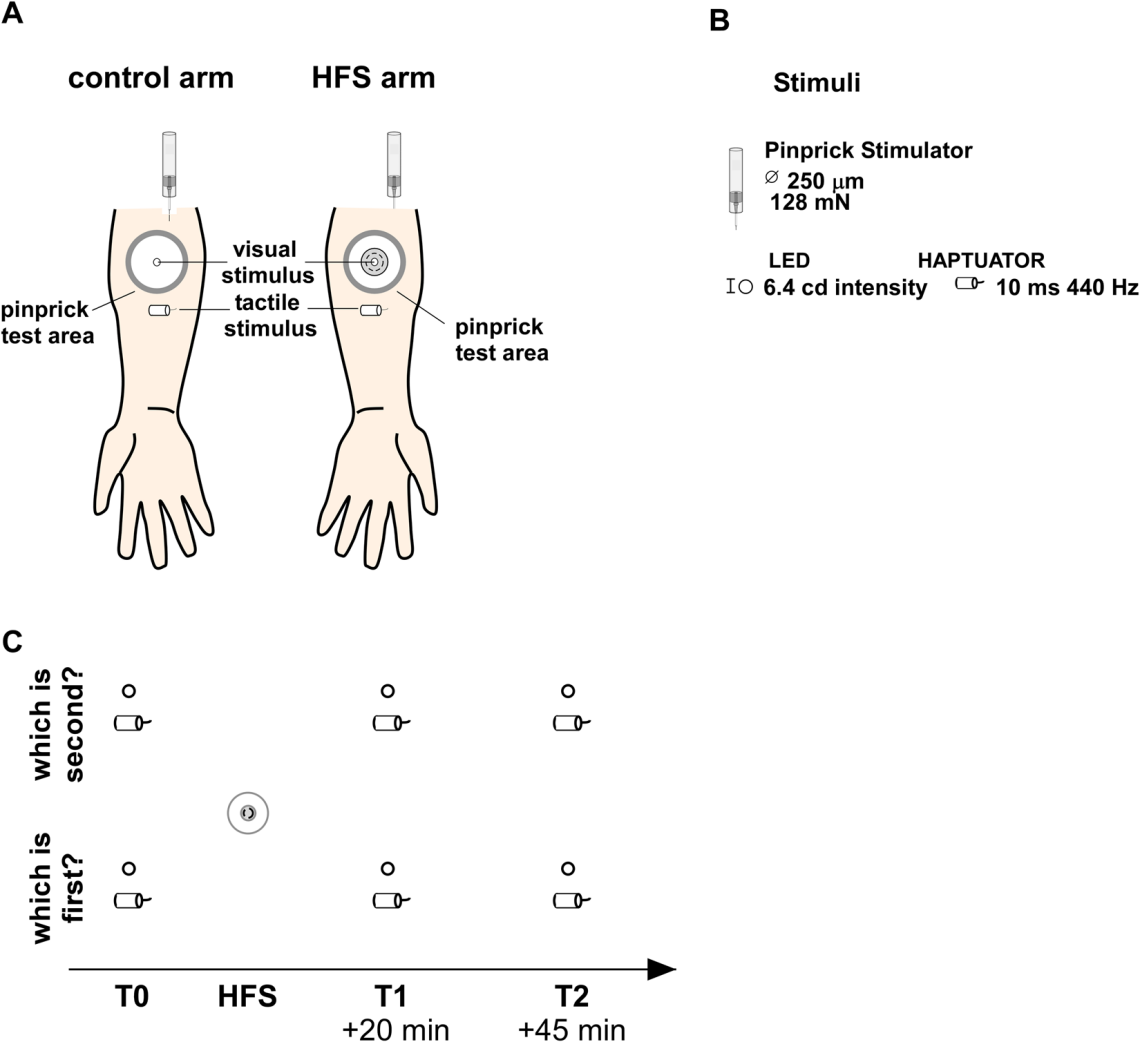
Experimental procedure. Panel A. Participants received, at different SOAs, pairs of either visual or vibrotactile stimuli on either forearm. Secondary hyperalgesia was tested by using pinprick stimuli. The location where visual and tactile stimuli were applied was established on the basis of previous studies^8,9^. Panel B. Details of the stimuli. Panel C. Participants were asked to report verbally which arm was perceived as stimulated first (or second). Tasks were performed before applying HFS (T0), 20 minutes (T1) and 45 minutes (T2) after HFS.

*Visual stimuli* were presented by means of two white light emitting diodes (LED) with a 17-lm luminous flux, a 6.40-cd luminous intensity, 5-ms duration and a 120° diffusion angle (GM5BW97330A, Sharp Corporation, Japan). They were perceived as brief flashes. LEDs were attached onto the arms by means of medical tape approximately 1 cm from the area of the volar forearm where HFS was applied and on the homologue area on the control arm. This location was chosen according to a previous study^9^ and on the basis of the average reported spread of secondary hyperalgesia^6^ (Fig. 3A). The exact location was marked on the skin at the beginning of the experimental procedure to ensure correct and constant positioning of the LEDs throughout the blocks. Before starting the experiment, we enquired whether stimuli were perceived as equally bright. In case of a negative response, the LED perceived as less intense was slightly moved along the medial line on the volar forearm until the stimuli were perceived as equally bright. A third yellow LED, equidistant from the arms at approximately 40 cm from the trunk (min. 0.7-cd luminous intensity at 20-mA, 120° diffusion angle, Multicomp, Farnell element14, UK) was used as fixation point during the task. At the end of each block we asked whether the stimuli were perceived as having the same luminosity during the block.

*Tactile stimuli* were generated by two vibrotactile transducers driven by standard audio amplifiers (TL-002-14R Haptuator Redesign, Tactile Labs, Inc., Montreal, Canada) secured to the arms by means of gauze. The stimuli lasted 10 ms, and were vibrating at 440 Hz. The stimulators were attached onto the arms approximately at 1.5 cm from the stimulated area, in line with previous studies^6,8^ (see also the comment for the visual stimuli). Also in this case, the exact location was marked on the skin at the beginning of the experiment and kept constant across the blocks. At the beginning of the experiment we checked whether the perceived intensity of the stimuli was the same between the arms. In case of a negative response, the haptuator perceived as less intense was slightly moved along the medial line on the volar forearm until the stimuli were perceived as equally intense.

*Secondary hyperalgesia* was induced by HFS of the skin using a specifically designed electrode made of 16 blunt stainless steel pins with a diameter of 0.2 mm protruding 1 mm from the base^3,60^. The pins were placed in a circle with a diameter of 10 mm and served as cathode. The stainless steel reference electrode, the anode, was placed surrounding the cathode and had an inner diameter of 22 mm and an outer diameter of 40 mm. HFS consisted of five trains of 100 Hz electrical stimuli (pulse width 2 ms) that lasted 1 second each and were repeated at a 10 s interval. The pulses were generated by a constant-current electrical stimulator (DS7A, Digitimer Ltd, Welwyn Garden City, UK). The duration of the whole procedure was 50 seconds. HFS was applied on the volar forearm skin of either the dominant or non-dominant arm, which was counterbalanced across participants. The intensity of stimulation was adjusted individually at 20 times the absolute detection threshold to a single pulse. The average threshold for the single pulse detection was 0.25 ± 0.09 mA, and the average intensity used for the sensitization procedure was 4.9 ± 1.8 mA.

### Procedure

The experiment was conducted in a dimly lit room. During the experiment, participants sat comfortably on a chair with their chin positioned on a chin rest in order to minimize head movement towards one of the arms. The arms were positioned palms up on a table in front of them. The distance between the arms was kept constant at approximately 30 cm. Visual and tactile stimuli were presented in pairs on either arm. At the beginning of the procedure, the experimenter who placed the haptuators and the LEDs on the skin was blind to the side of HFS stimulation.

At the beginning of the experiment, a familiarization phase composed of 4 short blocks (2 for the visual and 2 for the tactile TOJ) was run. In each block, 10 pairs of stimuli were presented for each modality. After familiarization, the actual experiment was started. The visual and tactile TOJs were performed in 4 separate blocks (2 for the visual and 2 for the tactile TOJ), each of which was composed of 40 trials. This number was chosen in accordance with previous studies having measured reliable parameters and having successively found PSS shifts using 20 to 40 trials^28,29,61–63^. A trial started with the illumination of the yellow fixation point. After 500 ms, the pair of stimuli was presented. During the familiarization phase, SOAs between the two stimuli were of ±145 and ±200 ms for the visual TOJ, and ±150 and ±200 ms for the the tactile TOJ. All participants correctly understood and performed the task. During the actual experiment twenty possible SOAs were defined for the visual TOJ task (±200, ±145, ±90, ±75, ±60, ±45, ±30, ±15, ±10, ±5 ms), and 22 for the tactile TOJ task (±400, ±200, ±150, ±90, ±75, ±60, ±45, ±30, ±15, ±10, ±5 ms). Negative values indicate that the left-sided stimulus was presented before the right-sided one, positive values indicate the reverse. The SOA was chosen at each trial on the basis of the participant’s performance in all previous trials, according to the adaptive PSI method^64^ (implemented through the Palamedes Toolbox^65^). The adaptive PSI method adopts a Bayesian framework where parameters of interest are estimated without probing extensively all the predefined SOAs an equal number of times. Starting from pre-defined priors relative to the threshold and the slope of the logistic function fitting the participant’s responses (that we set as 0 ± 20 for the threshold and 0.06 ± 0.6 for the slope; see^28^), the PSI algorithm updates at each trial the joint distribution of the slope and the threshold and chooses the subsequent SOA to minimize the expected entropy of the posterior distribution. In such a way, the number of trials needed to achieve a stable estimate of the parameters under exam can be reduced. In one block participants had to report verbally which side was perceived as stimulated first (‘*which is first?’*), in the other which was perceived as stimulated second (‘*which is second?*’). The condition ‘*which is second?*’ allows limiting the influence of response/decisional biases over perceptual biases^12,25,66^. No instruction was given regarding response speed. Participants’ responses were encoded manually by the experimenter and no feedback about the accuracy of the response was provided. A new trial started 2000 ms after the response was encoded.

The TOJ tasks were performed at three time points: before HFS (T0), 20 (T1) and 45 (T2) minutes after HFS. The order of the sensory modalities was counterbalanced across participants, the order ‘which is first’, ‘which is second’ was randomized.

### Measures

The presence of HFS-induced secondary hyperalgesia was assessed using a calibrated pinprick stimulator exerting a force of 128 mN (MRC Systems, Heidelberg, Germany). Participants were asked to rate the average sensation elicited by 3 consecutive stimuli applied at different locations onto the skin surrounding the area onto which HFS was applied and by 3 stimuli applied onto the homologue area of the contralateral non-sensitized arm. Ratings were provided on a Numerical Rating Scale (NRS) ranging from 0 to 100, and participants were informed that the number of 50 represented the transition from the non-painful to the painful domain of sensation. The extremes of the scale were described as ‘I did not feel anything’ (NRS = 0) and ‘pain as bad as I can imagine’ (NRS = 100). Half of the participants received the pinprick stimuli before the beginning of each block, the other after each block.

Data were fitted with a logistic function, which allowed us to derive the parameters of interest: the α and the β (as in^28^). The α defines the *threshold* of the function and characterizes in the present study the Point of Subjective Simultaneity (PSS) corresponding to the SOA at which two stimuli are reported as perceived first equally often. In the present study the logistic function was fitted on the probability of reporting the stimuli presented at the HFS side. Therefore, the PSS reflects the SOAs at which the probability of responding HFS as first stimulated arm equals 50%. The β defines the *slope* of the logistic function, namely, it describes the precision of the participants’ responses. Considering that the aim of this study was to investigate perceptual biases, our main outcome was the analysis of the threshold (the α), i.e. the PSS. Results of the slope (the β) are reported for completeness.

Figure 3 shows the experimental procedure.

### Statistical analysis

#### Frequentist analysis

Statistical analyses were conducted using IBM Statistics SPSS 19 (Armonk, NY: IBM 22 Corp.).

#### Mechanical hyperalgesia

The presence of secondary hyperalgesia was assessed by comparing the perceived intensity of the average of the three mechanical stimuli applied at each time point by means of a repeated-measure ANOVA with ‘*Time’* (T0, T1, T2) and *‘Side’* (control vs. HFS) as within-subject factors.

#### Perceptual bias

Before analyzing TOJ values, the PSS and slope values of the ‘*which is first?*’ and ‘*which is second?*’ conditions were averaged for each modality and each time period. In order to match the data of the two groups of participants according to the side of HFS the values of participants who received HFS onto the right arm were ‘flipped’, i.e. multiplied by -1. T-tests against zero were used to disclose significant perceptual biases (PSS significantly different from zero). A one-way repeated-measure ANOVA using the factor ‘*Time’* was then conducted, separately for each modality, to assess changes of PSS and slope values across time for each modality. Greenhouse-Geisser corrections of degrees of freedom were used in case of violation of sphericity. Effect sizes were estimated by means of partial Eta squared (η^2^_p_). Contrasts were used when necessary.

#### Bayesian analysis

Bayesian analysis on the PSS values were performed with JASP^67^. An advantage of the Bayesian approach over the frequentist approach consists in comparing the evidence for the null (H_0_) and alternative (H_1_) models, in our case that HFS did not lead to a perceptual bias (H_0_) and that HFS induced a bias (H_1_)^32,68^. The strength of evidence for one model or the other is quantified by the Bayes factor of B_10_ for H_1_ and B_01_ for H_0._ For instance, a BF_10_ of 7 indicates that the data are 7 times more likely under H_1_ than under H_0._ Conventionally, it is considered that values between 1 and 3 indicate ‘*anecdotal*’ evidence, from 3 to 10 ‘*moderate*’, from 10 to 30 ‘*strong*’ and above 30 ‘*very strong*’^69.^ Bayesian analyses were performed, as for the frequentist approach, for t-tests and repeated measure ANOVAs separately for modality. An additional 3 × 2 ANOVA, using both ‘*Time’* and ‘*Modality’* as factors was carried out to compare possible differential effects of HFS on the modality (see results section). Given the lack of a priori knowledge on the effect sizes, a default Cauchy prior was used (0.707). Results using wider priors are presented in the supplementary material. The datasets and the design are available on the Open Science Framework platform.

## Electronic supplementary material

Supplementary material

## References

[1] Loeser JD, Treede RD (2008). The Kyoto protocol of IASP Basic Pain Terminology. Pain.

[2] LaMotte RH, Shain CN, Simone DA, Tsai EF (1991). Neurogenic hyperalgesia: psychophysical studies of underlying mechanisms. Journal of neurophysiology.

[3] Klein T, Magerl W, Hopf HC, Sandkuhler J, Treede RD (2004). Perceptual correlates of nociceptive long-term potentiation and long-term depression in humans. The Journal of neuroscience: the official journal of the Society for Neuroscience.

[4] Pfau DB (2011). Analysis of hyperalgesia time courses in humans after painful electrical high-frequency stimulation identifies a possible transition from early to late LTP-like pain plasticity. Pain.

[5] Ali Z, Meyer RA, Campbell JN (1996). Secondary hyperalgesia to mechanical but not heat stimuli following a capsaicin injection in hairy skin. Pain.

[6] van den Broeke, E. N., Lenoir, C. & Mouraux, A. Secondary hyperalgesia is mediated by heat-insensitive A-fibre nociceptors. *The Journal of physiology* **594,** 6767–6776 (2016). 10.1113/JP272599 PMC5108905 27377467

[7] van den Broeke EN, Lambert J, Huang G, Mouraux A (2016). Central Sensitization of Mechanical Nociceptive Pathways Is Associated with a Long-Lasting Increase of Pinprick-Evoked Brain Potentials. Frontiers in human neuroscience.

[8] Van Den Broeke, E. N. & Mouraux, A. High frequency electrical stimulation of human skin induces heterotopical mechanical and heat hyperalgesia and enhanced responses to vibrotactile input. *Journal of neurophysiology* **111,** 1564–1573 (2014). 10.1152/jn.00651.2013 24453277

[9] Torta DM (2017). Intense pain influences the cortical processing of visual stimuli projected onto the sensitized skin. Pain.

[10] Hansson P (2014). Translational aspects of central sensitization induced by primary afferent activity: what it is and what it is not. Pain.

[11] Woolf CJ (2014). What to call the amplification of nociceptive signals in the central nervous system that contribute to widespread pain?. Pain.

[12] Shore DI, Spence C, Klein RM (2001). Visual prior entry. Psychological science.

[13] Vibell J, Klinge C, Zampini M, Spence C, Nobre AC (2007). Temporal order is coded temporally in the brain: early event-related potential latency shifts underlying prior entry in a cross-modal temporal order judgment task. Journal of cognitive neuroscience.

[14] van den Broeke EN (2012). The effect of high-frequency conditioning stimulation of human skin on reported pain intensity and event-related potentials. Journal of neurophysiology.

[15] van den Broeke EN, Mouraux A (2014). Enhanced brain responses to C-fiber input in the area of secondary hyperalgesia induced by high-frequency electrical stimulation of the skin. Journal of neurophysiology.

[16] van den Broeke EN, van Heck CH, van Rijn CM, Wilder-Smith OH (2011). Neural correlates of heterotopic facilitation induced after high frequency electrical stimulation of nociceptive pathways. Molecular pain.

[17] van den Broeke EN (2010). Neurophysiological correlates of nociceptive heterosynaptic long-term potentiation in humans. Journal of neurophysiology.

[18] Hillyard SA, Anllo-Vento L (1998). Event-related brain potentials in the study of visual selective attention. Proceedings of the National Academy of Sciences of the United States of America.

[19] Woldorff MG (1993). Modulation of early sensory processing in human auditory cortex during auditory selective attention. Proceedings of the National Academy of Sciences of the USA.

[20] Hillyard SA, Vogel EK, Luck SJ (1998). Sensory gain control (amplification) as a mechanism of selective attention: electrophysiological and neuroimaging evidence. Philosophical transactions of the Royal Society of London. Series B, Biological sciences.

[21] Mangun GR, Hillyard SA (1988). Spatial gradients of visual attention: behavioral and electrophysiological evidence. Electroencephalography and Clinical Neurophysiology.

[22] Van Voorhis S, Hillyard SA (1977). Visual evoked potentials and selective attention to points in space. Perception & psychophysics.

[23] Legrain V, Guerit JM, Bruyer R, Plaghki L (2002). Attentional modulation of the nociceptive processing into the human brain: selective spatial attention, probability of stimulus occurrence, and target detection effects on laser evoked potentials. Pain.

[24] Redden RS, d’Entremont G, Klein RM (2017). Further evidence in favor of prior entry from endogenous attention to a location in space. Attention, perception & psychophysics.

[25] Spence C, Parise C (2010). Prior-entry: a review. Consciousness and cognition.

[26] Heed T, Azanon E (2014). Using time to investigate space: a review of tactile temporal order judgments as a window onto spatial processing in touch. Frontiers in psychology.

[27] Titchener, E. B. *Lectures on the elementary psychology of feeling and attention*. (Macmillan, 1908).

[28] Filbrich L, Alamia A, Burns S, Legrain V (2017). Orienting attention in visual space by nociceptive stimuli: investigation with a temporal order judgment task based on the adaptive PSI method. Experimental brain research.

[29] Filbrich L, Alamia A, Blandiaux S, Burns S, Legrain V (2017). Shaping visual space perception through bodily sensations: Testing the impact of nociceptive stimuli on visual perception in peripersonal space with temporal order judgments. PloS one.

[30] Kingdom, F. & Prins, N. *Psychophysics: A practical introduction*. (Elsevier, 2010).

[31] van den Broeke EN, Geene N, van Rijn CM, Wilder-Smith OH, Oosterman J (2014). Negative expectations facilitate mechanical hyperalgesia after high-frequency electrical stimulation of human skin. European journal of pain.

[32] Wagenmakers, E. J. *et al*. Bayesian inference for psychology. Part I: Theoretical advantages and practical ramifications. *Psychonomic bulletin & review*10.3758/s13423-017-1343-3 (in press). 10.3758/s13423-017-1343-3 PMC5862936 28779455

[33] Masson ME (2011). A tutorial on a practical Bayesian alternative to null-hypothesis significance testing. Behavior research methods.

[34] Zampini M (2005). Audiotactile temporal order judgments. Acta psychologica.

[35] Zampini M, Shore DI, Spence C (2005). Audiovisual prior entry. Neuroscience letters.

[36] Spence C, Shore DI, Klein RM (2001). Multisensory prior entry. Journal of experimental psychology. General.

[37] Moseley GL, Gallace A, Spence C (2009). Space-based, but not arm-based, shift in tactile processing in complex regional pain syndrome and its relationship to cooling of the affected limb. Brain: a journal of neurology.

[38] De Paepe AL, Crombez G, Spence C, Legrain V (2014). Mapping nociceptive stimuli in a peripersonal frame of reference: Evidence from a temporal order judgment task. Neuropsychologia.

[39] Van Damme S, Gallace A, Spence C, Crombez G, Moseley GL (2009). Does the sight of physical threat induce a tactile processing bias? Modality-specific attentional facilitation induced by viewing threatening pictures. Brain research.

[40] Vanden Bulcke C, Van Damme S, Durnez W, Crombez G (2013). The anticipation of pain at a specific location of the body prioritizes tactile stimuli at that location. Pain.

[41] Vanden Bulcke C, Crombez G, Durnez W, Van Damme S (2015). Is attentional prioritization on a location where pain is expected modality-specific or multisensory?. Consciousness and cognition.

[42] Yates MJ, Nicholls ME (2009). Somatosensory prior entry. Attention, perception & psychophysics.

[43] De Paepe AL, Crombez G, Legrain V (2015). From a Somatotopic to a Spatiotopic Frame of Reference for the Localization of Nociceptive Stimuli. PloS one.

[44] Van der Burg E, Olivers CN, Bronkhorst AW, Theeuwes J (2008). Audiovisual events capture attention: evidence from temporal order judgments. Journal of vision.

[45] Stelmach LB, Herdman CM (1991). Directed attention and perception of temporal order. Journal of experimental psychology. Human perception and performance.

[46] Petersen SE, Posner MI (2012). The attention system of the human brain: 20 years after. Annual review of neuroscience.

[47] Torta DM, Legrain V, Mouraux A, Valentini E (2017). Attention to pain! A neurocognitive perspective on attentional modulation of pain in neuroimaging studies. Cortex; a journal devoted to the study of the nervous system and behavior.

[48] Rosenberg MD, Finn ES, Scheinost D, Constable RT, Chun MM (2017). Characterizing Attention with Predictive Network Models. Trends in cognitive sciences.

[49] Raz A, Buhle J (2006). Typologies of attentional networks. Nature reviews. Neuroscience.

[50] Takahashi T, Kansaku K, Wada M, Shibuya S, Kitazawa S (2013). Neural correlates of tactile temporal-order judgment in humans: an fMRI study. Cerebral cortex.

[51] Bressler DW, Fortenbaugh FC, Robertson LC, Silver MA (2013). Visual spatial attention enhances the amplitude of positive and negative fMRI responses to visual stimulation in an eccentricity-dependent manner. Vision research.

[52] Gandhi SP, Heeger DJ, Boynton GM (1999). Spatial attention affects brain activity in human primary visual cortex. Proceedings of the National Academy of Sciences of the United States of America.

[53] Somers DC, Dale AM, Seiffert AE, Tootell RB (1999). Functional MRI reveals spatially specific attentional modulation in human primary visual cortex. Proceedings of the National Academy of Sciences of the United States of America.

[54] Braga RM, Wilson LR, Sharp DJ, Wise RJ, Leech R (2013). Separable networks for top-down attention to auditory non-spatial and visuospatial modalities. NeuroImage.

[55] Corbetta M, Shulman GL (2002). Control of goal-directed and stimulus-driven attention in the brain. Nature Review Neuroscience.

[56] Shulman GL (1997). Top-down modulation of early sensory cortex. Cerebral cortex.

[57] Goltz D, Pleger B, Thiel SD, Villringer A, Muller MM (2013). Sustained spatial attention to vibrotactile stimulation in the flutter range: relevant brain regions and their interaction. PloS one.

[58] van den Broeke EN, de Vries B, Lambert J, Torta DM, Mouraux A (2017). Phase-locked and non-phase-locked EEG responses to pinprick stimulation before and after experimentally-induced secondary hyperalgesia. Clinical neurophysiology: official journal of the International Federation of Clinical Neurophysiology.

[59] Nicholls ME, Thomas NA, Loetscher T, Grimshaw GM (2013). The Flinders Handedness survey (FLANDERS): a brief measure of skilled hand preference. Cortex; a journal devoted to the study of the nervous system and behavior.

[60] Biurrun Manresa JA, Morch CD, Andersen OK (2010). Long-term facilitation of nociceptive withdrawal reflexes following low-frequency conditioning electrical stimulation: a new model for central sensitization in humans. European journal of pain.

[61] Vanderclausen C, Filbrich L, Alamia A, Legrain V (2017). Investigating peri-limb interaction between nociception and vision using spatial depth. Neuroscience letters.

[62] Filbrich L (2017). Biased visuospatial perception in complex regional pain syndrome. Scientific reports.

[63] Filbrich, L., Halicka, M., Alamia, A. & Legrain, V. Investigating the spatial characteristics of the crossmodal interaction between nociception and vision using gaze direction. *Consciousness and cognition* **57**, 106–115 (2018). 10.1016/j.concog.2017.11.011 29207312

[64] Kontsevich LL, Tyler CW (1999). Bayesian adaptive estimation of psychometric slope and threshold. Vision research.

[65] Prins, N. & Kingdon, F. Palamedes: Matlab routines for analyzing psychophysical data. *Palamedes: matlab routines for analyzing psychophysical data* (2009).

[66] Scharlau I (2004). Evidence against response bias in temporal order tasks with attention manipulation by masked primes. Psychological research.

[67] JASP Version 0.8.4 (2017).

[68] Wagenmakers, E. J. *et al*. Bayesian inference for psychology. Part II: Example applications with JASP. *Psychonomic bulletin & review* (2017). 10.3758/s13423-017-1323-7 PMC5862926 28685272

[69] Jeffreys, H. *Theory of probability (3rd ed.)*. (Oxford University Press, 1961).

