# Supplementary material for: No perceptual prioritization of non-nociceptive vibrotactile and visual stimuli presented on a sensitized body part
Source: Sci Rep. 2018 Mar 29;8:5359. doi: 10.1038/s41598-018-23135-6 (PMC5876401; doi:10.1038/s41598-018-23135-6)
Supplement: Supplementary file 1 — Supplementary material [file 41598_2018_23135_MOESM1_ESM.docx]

**No perceptual prioritization of non-nociceptive vibrotactile and visual stimuli presented on a sensitized body part**

Torta DM*, Filbrich L*, Van Den Broeke EN, Legrain V

**Supplementary material**

**Bayesian T-Test**

| Bayesian One Sample T-Test | | | | | |
| --- | --- | --- | --- | --- | --- |
| Tactile | | | | | |
|  | | **BF₀₁** | | **error %** | |
| T0 |  | 2.924 |  | 8.932e -5 |  |
| T1 |  | 4.102 |  | 1.213e -4 |  |
| T2 |  | 3.964 |  | 1.206e -4 |  |
| Visual | | | | | |
| T0 |  | 3.338 |  | 1.091e -4 |  |
| T1 |  | 1.988 |  | 2.542e -5 |  |
| T2 |  | 4.081 |  | 1.212e -4 |  |

Table S1. Results of the Bayesian one sample T-test for the tactile and visual TOJs. BF_01_ refers to the evidence that the data provide for H_0_ versus H_1_, it represents the inverse of the Bayes factor BF_10_ (B_01_=1/BF_10_). The highest the value of B01, the more evidence there is for the null-hypothesis.

**Inferential Plots Tactile: T0**

**(A) Prior and Posterior (B) Bayes Factor Robustness Check**


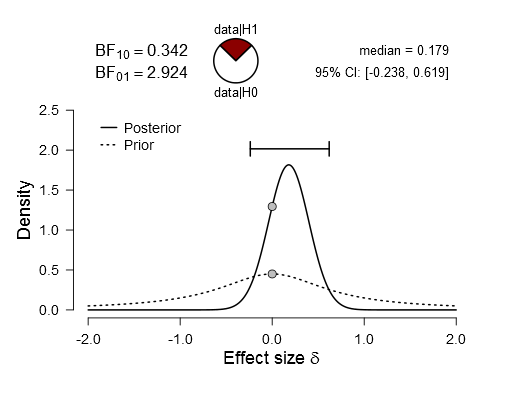

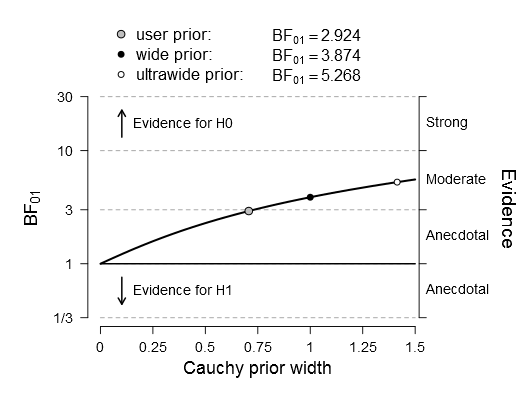


Figure S1. Left panel (A). Evidence in favor of the H1 (BF_10_) and of the H0 (BF_01_), and prior and posterior distributions. Right panel (B): evidence in favor of BF_01_ as a function of the Cauchy prior width. The Cauchy prior represents the median of the alternative hypothesis effect size distribution.

**Inferential Plots Tactile: T1**

**(A) Prior and Posterior (B) Bayes Factor Robustness Check**


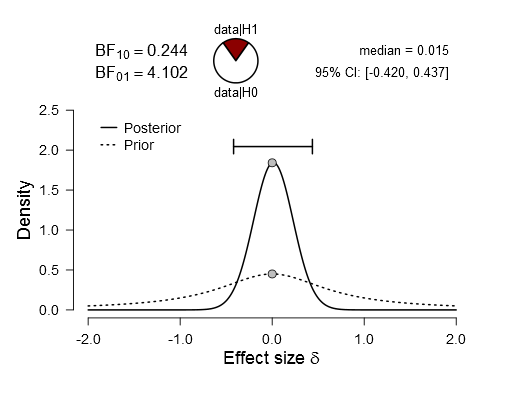

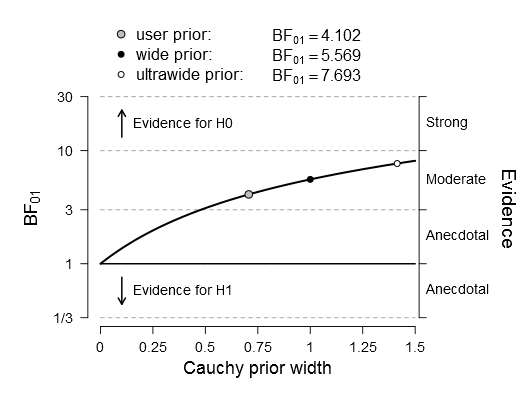


 Figure S2. Left panel (A). Evidence in favor of the H1 (BF_10_) and of the H0 (BF_01_), and prior and posterior distributions. Right panel (B): evidence in favor of BF_01_ as a function of the Cauchy prior width. The Cauchy prior represents the median of the alternative hypothesis effect size distribution.

**Inferential Plots Tactile: T2**

**(A) Prior and Posterior (B) Bayes Factor Robustness Check**


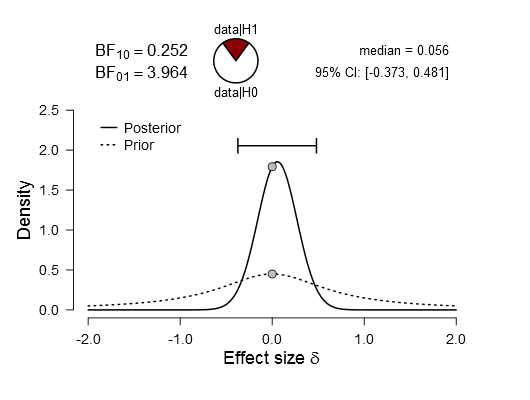

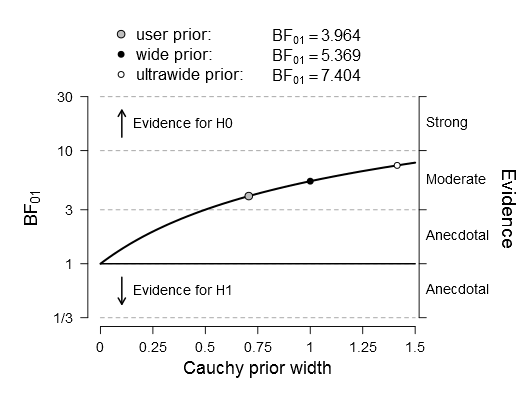


Figure S3. Left panel (A). Evidence in favor of the H1 (BF_10_) and of the H0 (BF_01_), and prior and posterior distributions. Right panel (B): evidence in favor of BF_01_ as a function of the Cauchy prior width. The Cauchy prior represents the median of the alternative hypothesis effect size distribution

**Inferential Plots Visual: T0**

**(A) Prior and Posterior (B) Bayes Factor Robustness Check**


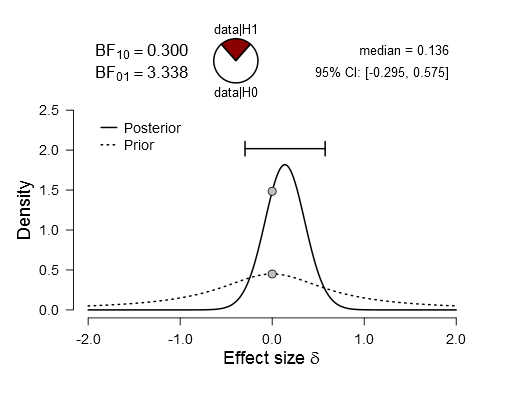

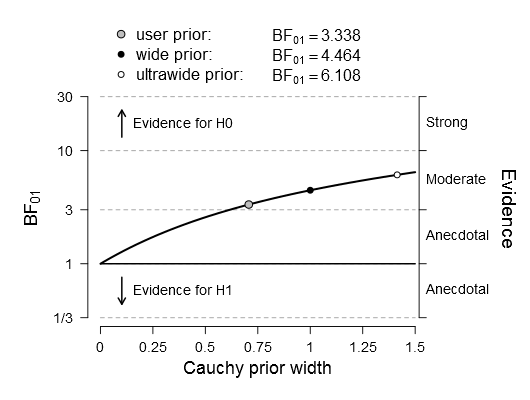


Figure S4. Left panel (A). Evidence in favor of the H1 (BF_10_) and of the H0 (BF_01_), and prior and posterior distributions. Right panel (B): evidence in favor of BF_01_ as a function of the Cauchy prior width. The Cauchy prior represents the median of the alternative hypothesis effect size distribution

**Inferential Plots Visual: T1**

**(A) Prior and Posterior (B) Bayes Factor Robustness Check**


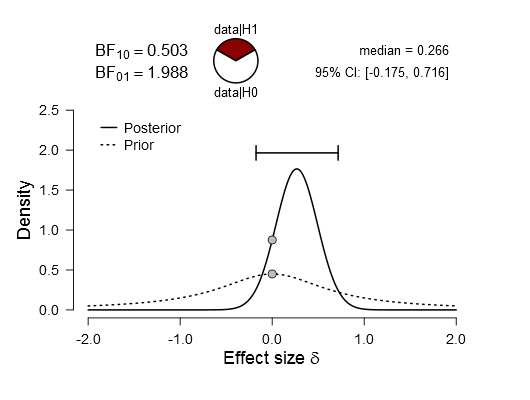

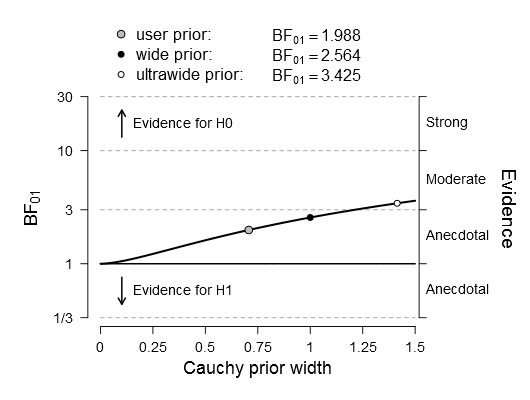


 Figure S5. Left panel (A). Evidence in favor of the H1 (BF_10_) and of the H0 (BF_01_), and prior and posterior distributions. Right panel (B): evidence in favor of BF_01_ as a function of the Cauchy prior width. The Cauchy prior represents the median of the alternative hypothesis effect size distribution

**Inferential Plots Visual: T2**

**(A) Prior and Posterior (B) Bayes Factor Robustness Check**


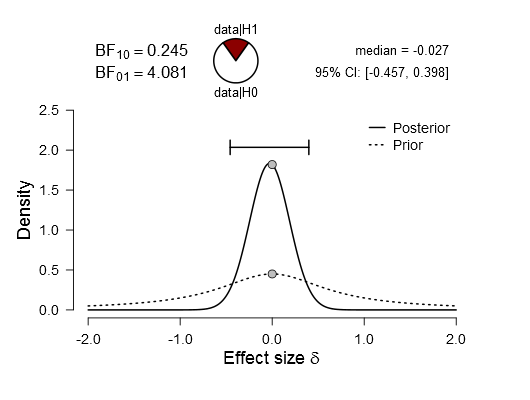

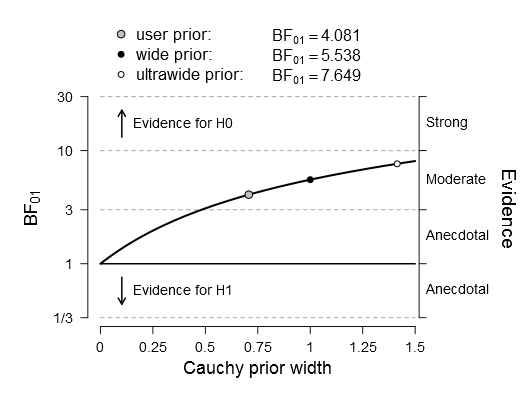


Figure S6. Left panel (A). Evidence in favor of the H1 (BF_10_) and of the H0 (BF_01_), and prior and posterior distributions. Right panel (B): evidence in favor of BF_01_ as a function of the Cauchy prior width. The Cauchy prior represents the median of the alternative hypothesis effect size distribution

**Bayesian Repeated Measures 2 x 3 ANOVA**

| Model Comparison | | | | | | | | | | | |
| --- | --- | --- | --- | --- | --- | --- | --- | --- | --- | --- | --- |
| Models | | **P(M)** | | **P(M\|data)** | | **BF _01_** | | **BF _10_** | | **error %** | |
| Null model (incl. subject) |  | 0.200 |  | 0.729 |  | 10.777 |  | 1.000 |  |  |  |
| Modality |  | 0.200 |  | 0.148 |  | 0.692 |  | 0.202 |  | 1.431 |  |
| Time |  | 0.200 |  | 0.098 |  | 0.433 |  | 0.134 |  | 1.257 |  |
| Modality + Time |  | 0.200 |  | 0.020 |  | 0.080 |  | 0.027 |  | 2.072 |  |
| Modality + Time + Modality  ✻  Time |  | 0.200 |  | 0.006 |  | 0.024 |  | 0.008 |  | 1.513 |  |
|  | | | | | | | | | | | |
| *Note.*  All models include subject. | | | | | | | | | | | |

Table S2. Results of the Bayesian 2 x 3 ANOVA having as factors the *Time* (T0, T1, and T2) and the *Modality* (Visual and Tactile). BF_01_ refers to the evidence that the data provide for H_0_ versus H_1_, it represents the inverse of the Bayes factor BF_10_ (B_01_=1/BF_10_). The highest the value of B01, the more evidence there is for the null-hypothesis.

**Frequentist Repeated Measures 2 x 3 ANOVA**

|  | | | | | | | | | | | |  |
| --- | --- | --- | --- | --- | --- | --- | --- | --- | --- | --- | --- | --- |
|  | | **Sum of Squares** | | **df** | | **Mean Square** | | **F** | | **p** | | **Partial eta squared** |
| Modality |  | 0.257 |  | 1 |  | 0.257 |  | 0.001 |  | 0.979 |  | 0.000 |
| Residual |  | 6316.300 |  | 17 |  | 371.547 |  |  |  |  |  |  |
| Time |  | 163.198 |  | 2 |  | 81.599 |  | 0.621 |  | 0.543 |  | 0.035 |
| Residual |  | 4467.527 |  | 34 |  | 131.398 |  |  |  |  |  |  |
| Modality ✻ Time |  | 318.574 |  | 2 |  | 159.287 |  | 2.423 |  | 0.104 |  | 0.125 |
| Residual |  | 2235.069 |  | 34 |  | 65.737 |  |  |  |  |  |  |

*Type 3 sum of square*

Table S3. Results of the Frequentist 2 x 3 ANOVA having as factors the *Time* (T0, T1, and T2) and the *Modality* (Visual and Tactile).
